# ESR Essentials: response assessment criteria in oncologic imaging—practice recommendations by the European Society of Oncologic Imaging

**DOI:** 10.1007/s00330-024-11006-w

**Published:** 2024-08-13

**Authors:** Giulia A. Zamboni, Giovanni Cappello, Damiano Caruso, Sofia Gourtsoyianni, Clemens Cyran, Heinz-Peter Schlemmer, Melvin D’Anastasi, Laure Fournier, Emanuele Neri

**Affiliations:** 1 Department of Diagnostics and Public Health, Institute of Radiology, University of Verona, Policlinico GB Rossi, P.Le LA Scuro 10, 37134 Verona, Italy; 2 Radiology Unit, Candiolo Cancer Institute, FPO-IRCCS, Str. Prov.le 142 km 3.95, 10060 Candiolo (Turin), Italy; 3 Department of Medical Surgical Sciences and Translational Medicine, Sapienza University of Rome, Sant’Andrea University Hospital, Via Di Grottarossa, 1035-1039, 00189 Rome, Italy; 4 1st Department of Radiology, School of Medicine, National and Kapodistrian University of Athens, Areteion Hospital, 76, Vas. Sophias Ave., 11528 Athens, Greece; 5 Department of Radiology, LMU University Hospital, LMU Munich, Marchioninistr. 15, 81377 Munich, Germany; 6 Department of Radiology, German Cancer Research Center (DKFZ), Im Neuenheimer Feld 280, 69120 Heidelberg, Germany; 7 Medical Imaging Department, Mater Dei Hospital, University of Malta, Msida, 2090 MSD Malta; 8 Université Paris Cité, AP-HP, Hôpital Européen Georges Pompidou, Department of Radiology, PARCC UMRS 970, INSERM, Paris, France; 9 Department of Translational Research, Academic Radiology, University of Pisa, 56124 Pisa, Italy

**Keywords:** Response evaluation criteria in solid tumors, Tomography (x-ray computed), Neoplasms, Neoplasm metastasis, Biomarkers

## Abstract

**Abstract:**

Assessing the response to oncological treatments is paramount for determining the prognosis and defining the best treatment for each patient. Several biomarkers, including imaging, can be used, but standardization is fundamental for consistency and reliability. Tumor response evaluation criteria have been defined by international groups for application in pharmaceutical clinical trials evaluating new drugs or therapeutic strategies. RECIST 1.1 criteria are exclusively based on unidimensional lesion measurements; changes in tumor size are used as surrogate imaging biomarkers to correlate with patient outcomes. However, increased tumor size does not always reflect tumor progression. The introduction of immunotherapy has led to the development of new criteria (iRECIST, Level of Evidence (LoE) Ib) that consider the possibility that an increase in disease burden is secondary to the immune response instead of progression, with the new concept of Unconfirmed Progressive Disease (a first progression event which must be confirmed on follow-up). Specific criteria were devised for HCC (mRECIST, LoE IV), which measure only enhancing HCC portions to account for changes after local therapy. For GIST treated with imatinib, criteria were developed to account for the possible increase in size reflecting a response rather than a progression by assessing both tumor size and density on CT (Choi, LoE II). This article provides concise and relevant practice recommendations aimed at general radiologists to help choose and apply the most appropriate criteria for assessing response to treatment in different oncologic scenarios. Though these criteria were developed for clinical trials, they may be applied in clinical practice as a guide for day-to-day interpretation.

**Key Points:**

- *Response evaluation criteria, designed for use in clinical trials, might serve as a surrogate biomarker for overall survival*.
- *RECIST 1.1 defines measurable and non-measurable disease among which target lesions and non-target lesions are selected at baseline as reference for follow-ups*.
- *Some therapies and/or cancers require the use of different criteria, such as iRECIST, mRECIST, and Choi criteria*.

## Key recommendations

- Response evaluation criteria should be used in pharmaceutical clinical trials testing new drugs or treatment strategies. They may, however, also be used in clinical practice as a framework guiding image interpretation to provide consistent evaluations of tumor response across readers.
- RECIST 1.1 criteria should be used for solid tumors treated by systemic therapies and remain the reference standard in pharmaceutical clinical trials (level of evidence Ib).
- Specific criteria should be used for some tumors/therapies. iRECIST are exploratory criteria that should be collected in addition to RECIST 1.1 in clinical trials evaluating immune-based therapies (level of evidence Ib). mRECIST should be used to evaluate hepatocellular carcinoma (HCC) treated by focal or targeted therapies (level of evidence IV). Choi criteria should be used on CT to evaluate the response of gastrointestinal stromal tumors (GIST) to imatinib exclusively (level of evidence II).

## Introduction

**Fig. 1:**
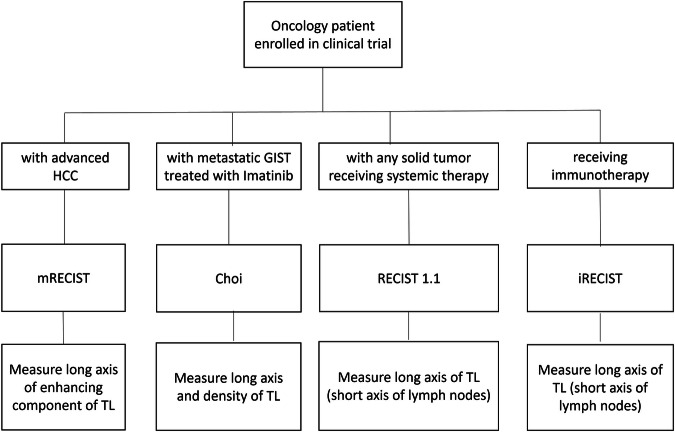
Flowchart on selecting the appropriate reporting criteria depending on the disease and type of treatment in patients with solid tumors enrolled in pharmaceutical treatment clinical trials

Assessing treatment response represents an essential crossroad in oncology patient management as it establishes whether a specific treatment has been effective. In oncological trials, the most important and accurate indicator of treatment effectiveness is patient overall survival (OS) [1]. Unfortunately, identifying statistically significant differences in survival is costly as it requires extended follow-up. Additionally, patients will often undergo more lines of treatment, so it is important to assess the impact of the different lines of treatment. Various biomarkers are used as surrogate measures of OS [2], including imaging biomarkers. However, imaging response to a specific treatment and subsequent change in overall tumor burden can serve as surrogate endpoints only if based on standardized, widely accepted, and easily applicable evaluation criteria [3]. The World Health Organization (WHO) published the first radiological response evaluation criteria in 1981 based on measuring tumor diameters [4]. Since then, several criteria have been developed and adapted for specific tumor types and anticancer treatments [3, 5]. Imaging plays an integral part in oncological clinical trial design, and it is up to radiologists to be able to apply appropriate response criteria effectively during imaging interpretation (Fig. 1).

## RECIST 1.1 criteria

Response evaluation criteria in solid tumors (RECIST) version 1.0, developed by the European Organisation for Research and Treatment of Cancer (EORTC), replaced WHO criteria in 2000 [6], followed by the upgraded RECIST version 1.1 in 2009, which are now widely used to evaluate the efficacy of treatment in patients with solid tumors enrolled in pharmaceutical clinical trials evaluating systemic therapies [7] and used less consistently in daily clinical practice of oncologic institutions [8]. RECIST 1.1 criteria are exclusively based on unidimensional lesion measurements, and changes in tumor size are used as surrogate imaging biomarkers to correlate with patient outcomes.

### Measurable/non-measurable and target/non-target lesions at baseline

An initial assessment (baseline) must be performed within the 4 weeks prior to treatment initiation. At baseline, tumor burden is divided into:

- Measurable lesions: lesions with longest diameter ≥ 10 mm, pathological lymph nodes with short axis ≥ 15 mm, and osteolytic/mixed lesions only if associated with solid tissue ≥ 10 mm;
- Non-measurable lesions: lesions with longest diameter of < 10 mm, pathological lymph nodes with short axis ≥ 10 mm and < 15 mm, osteoblastic lesions, and every tumor site that is objectively difficult to measure (e.g., ascites, pleural or pericardial effusion, leptomeningeal disease, lymphangitic involvement of the lung or skin, diffuse infiltrative type gastric cancer). Subsequently, target lesions (TL) and non-target lesions (NTL) must be chosen. Radiologists must select from the measurable lesions a maximum of five TL (up to two per organ), which must be representative of all affected organs and easily reproducible across time points. The longest diameter of each lesion is measured, preferably on the axial plane. For lymph nodes, the short axis is measured. Cystic metastases may be included among the TL (non-cystic metastases are preferred if present), while cavitating lesions should not be considered TL. The sum of the longest diameters (SLD) of the selected TLs signifies the starting point for evaluating response to treatment. The remaining measurable and all non-measurable lesions represent NTL and are only recorded and not measured.

### Follow-up examinations and assessment of treatment response

**Fig. 2:**
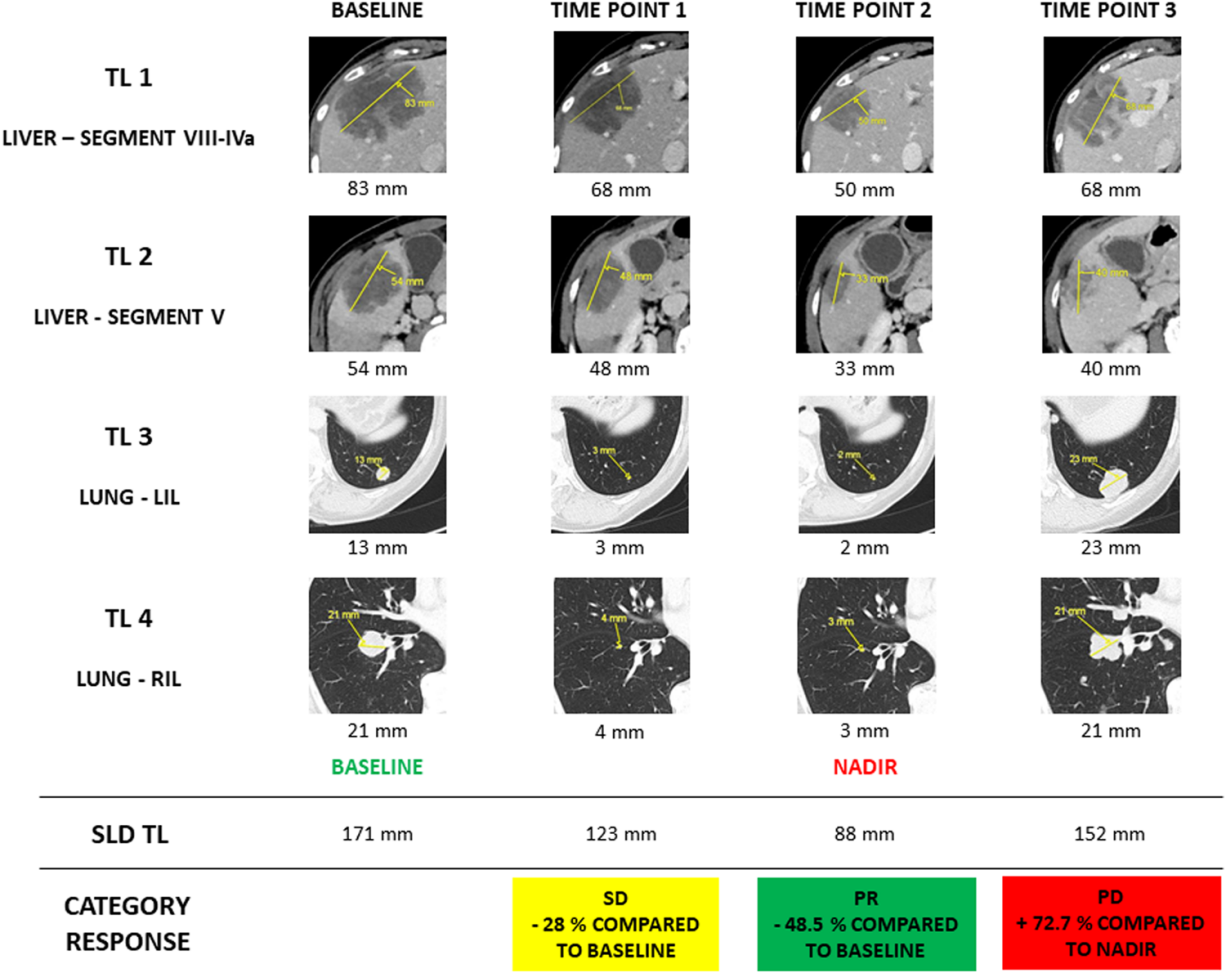
A 58-year-old female patient with metastatic colorectal cancer treated according to the chemotherapy scheme combination FOLFOXIRI. On baseline, metastatic disease included liver and lung disease and four TL were selected (maximum two per organ). According to RECIST 1.1 criteria, the percentage variation of TL SLD will determine the response category of TL. The first time point after 3 months of therapy showed a reduction of the TL SLD of 28% compared to baseline, and the assigned response to the treatment category for TL was SD (the threshold for PR is a reduction of 30% compared to baseline). The second time point after 6 months of therapy showed a further reduction of TL SLD compared to baseline (45.6%, PR). The third time point after 9 months of therapy showed an increase of TL SLD of 63.4% compared to nadir and therefore the assigned response to the treatment category for TL was PD (the threshold for PD is an increase of 20% compared to nadir). Baseline: imaging examination performed before the start of treatment; nadir: time point with the smallest SLD of target lesions. SD, stable disease; SLD, sum of the longest diameters; PD, progressive disease; PR, partial response; TL, target lesions

The TL and NTL selected at baseline must be re-assessed at all time points. Every TL must be measured when possible. When the lesion has disappeared, a 0 measurement should be logged. If the lesion is still present, if feasible, it should be measured even if very small. If the lesion is present but hard to measure, a 5 mm default diameter will be assigned. The short axis of pathological lymph nodes must be measured even if < 10 mm and added to the SLD of TL. The percentage variation of the SLD from the baseline or from the nadir (i.e., the time point with the smallest SLD) will determine the response category; PD requires an absolute increase in SLD of ≥ 5 mm (Table 1; Fig. 2).

**Table 1:** Response category of target lesions (TL), non-target lesions (NTL) and new lesions (NL)

Response category for target lesions	RECIST 1.1	iRECIST	mRECIST^a^	Choi
Complete response (CR)	Disappearance of all target lesions (lymph nodes must have a short axis < 10 mm)	Id. RECIST 1.1	Disappearance of any intratumoral arterial enhancement in target lesions	Id. RECIST 1.1
Partial response (PR)	Decrease of SLD of target lesions ≥ 30% compared to baseline	Id. RECIST 1.1	≥ 30% of SLD of viable portions (enhancement on arterial phase) of target lesions compared to baseline	A decrease in SLD of target lesions ≥ 10% or a decrease in tumor density (HU) ≥ 15% on CT compared to baseline
Stable disease (SD)	Neither sufficient shrinkage (compared to baseline) to qualify for PR nor sufficient increase (compared to nadir) to qualify for PD	Id. RECIST 1.1	Id. RECIST 1.1	Id. RECIST 1.1
Progressive disease (PD)	Increase of SLD of target lesions ≥ 20% (and ≥ 5 mm) compared to nadir	The first occurrence of progression will be defined as immune unconfirmed progressive disease (iUPD) and will need to be confirmed by a second progression event, defined as either a further progression in the same lesion type (TL, NTL or NL) or in a new lesion type	Increase of SLD of viable (enhancing) portions of target lesions ≥ 20% compared to nadir	Increase of SLD of target lesions ≥ 10% and does not meet criteria of PR by tumor density (HU) on CT

Response category for non-target lesions	Definition			
Complete response (CR)	Disappearance of all non-target lesions (lymph nodes must have a short axis < 10 mm)	Id. RECIST 1.1	Id. RECIST 1.1	Id. RECIST 1.1
Non-complete response/ non-progressive disease (non-CR/non-PD)	Persistence of ≥ 1 non-target lesions	Id. RECIST 1.1	Id. RECIST 1.1	No obvious progression of non-measurable disease
Progressive disease (PD)	Unequivocal progression of existing non-target lesions. The increase of a single non-target lesion is not enough to determine a PD, but it is necessary to have unequivocal worsening in non-target disease, which would require a change in therapy (even in the presence of SD or PR in target disease)	The first occurrence of progression will be defined as immune unconfirmed progressive disease (iUPD) and will need to be confirmed by a second progression event, defined as either a further progression in the same lesion type (TL, NTL or NL) or in a new lesion type	Id. RECIST 1.1	New intratumoral nodules or increase in the size of the existing intratumoral nodules

Response category for new lesions	Definition			
Yes	Any new lesion for which the metastatic nature is certain.	The first occurrence of progression will be defined as immune unconfirmed progressive disease (iUPD) and will need to be confirmed by a second progression event, defined as either a further progression in the same lesion type (TL, NTL or NL) or in a new lesion type	Any new lesion for which the diagnosis of HCC or the metastatic nature is certain.	Any new lesion for which the metastatic nature is certain.
No	No new lesions	No new lesions	No new lesions	No new lesions

RECIST should be used in drug clinical trials, iRECIST 1.1 only if one of the drugs is an immunotherapy. mRECIST is applied when evaluating HCC, and Choi for GIST under imatinib (a targeted therapy)

^a^ For mRECIST, HCC are evaluated differently from all other metastatic sites. Target HCC lesions are defined as lesions with a nodular (clear boundaries, non-infiltrating) enhancement on arterial phase on CT or MRI with longest diameter ≥ 10 mm. For other metastatic sites, the same definition as RECIST 1.1 is applied. Non-target HCC lesions are defined if the HCC lesion is too small (< 10 mm), infiltrating or presents atypical enhancement (non-arterial). For other metastatic sites, the same definition as RECIST 1.1 is applied

The variation of the sum of the longest diameters (SLD) of the target lesions will determine the response category, while only a qualitative assessment will determine the response category of non-target lesions

Variation in NTLs is evaluated qualitatively, and they are described as present, disappeared, or in unequivocal progression. It is important to note that partial response does not apply to NTLs. The subsequent response categories are reported in Table 1.

The appearance of new metastatic lesions automatically determines progressive disease. In case of uncertain findings, treatment should be continued and specific findings evaluated at the following time point: if a new lesion(s) is confirmed, the imaging study in which the finding was first identified will become the time point of disease progression. Finally, for each time point, the overall response category is assigned by assessing TL + LNT ± new lesions (Table 2). This category response with the duration of response will be used to extrapolate the OS surrogate endpoints (e.g., progression-free survival, time to progression, objective response rate, best overall response).

**Table 2:** Overall response to treatment categories according to RECIST 1.1 criteria, which results from the fusion of the single category response (target lesions and non-target lesions) ± the presence of new lesion(s)

Target lesion response	Non-target lesion response	New lesions	Overall response of the time point
CR	CR	No	CR
CR	Non-CR/non-PD	No	PR
CR	NE	No	PR
PR	Non-PD or NE	No	PR
SD	Non-PD or NE	No	SD
NE	Non-PD	No	NE
PD	Any	Any	PD
Any	PD	Any	PD
Any	Any	Yes	PD

*CR* complete response, *NE* not evaluated, *Non-CR/non-PD* non-complete response/non-progressive disease, *SD* stable disease, *PD* progressive disease, *PR* partial response

### Limitation of RECIST 1.1 criteria

**Fig. 3:**
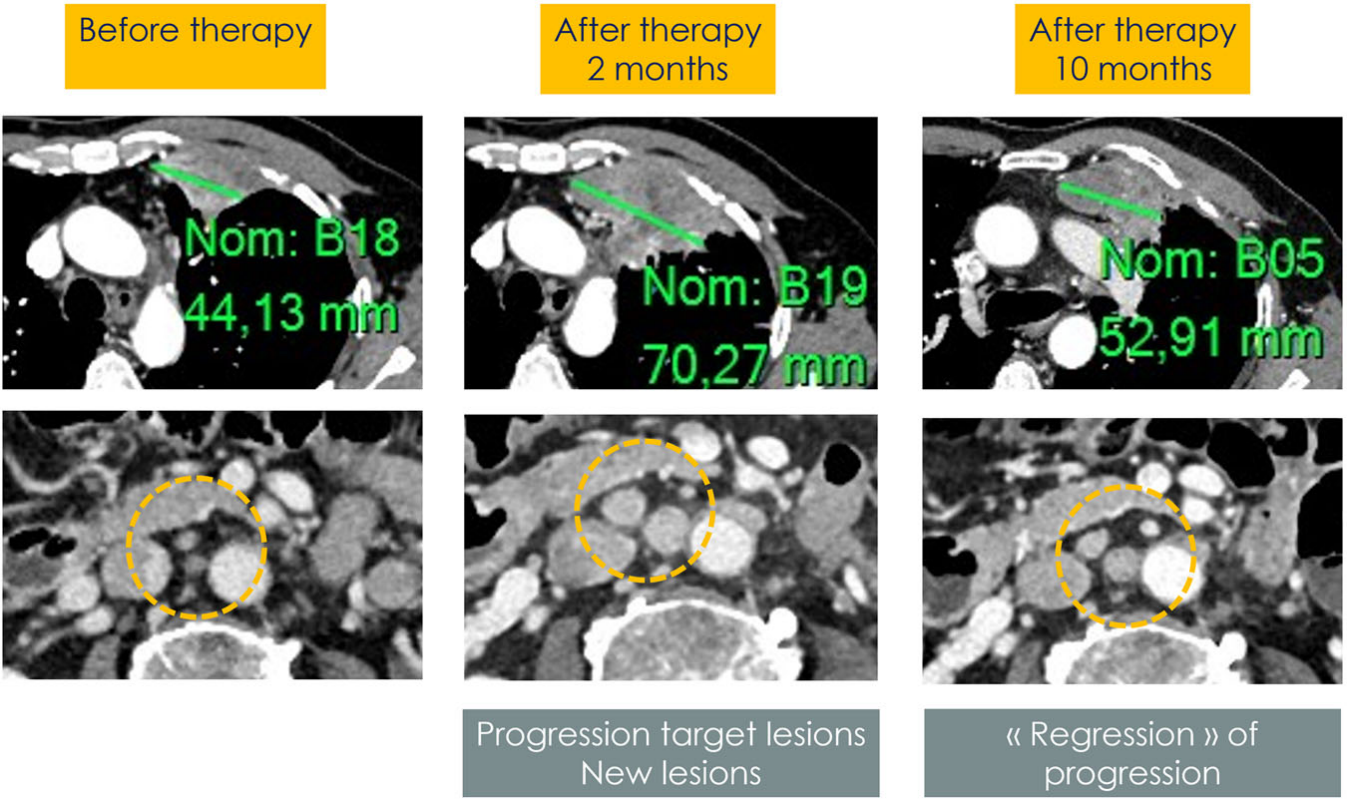
A 63-year-old male patient with metastatic ccRCC. On baseline, metastatic disease included pleural disease, among which one TL was selected (top left). There were no enlarged paraaortic lymph nodes (bottom left). The first time point 2 months after the start of immunotherapy (nivolumab) showed a significant size increase in TL and new lesions in the form of enlarged retroperitoneal lymph nodes (respectively top and bottom middle), resulting in iUPD. The next time point 2 months after the start of immunotherapy showed a subsequent decrease in the size of the TL and the normalization in the size of the lymph nodes. Disease progression was not confirmed, and the patient response was iSD, defining a pseudoprogression

Despite the widespread utilization of RECIST 1.1 criteria, concerns persist regarding the sole reliance on changes in tumor size. Several studies have demonstrated how inter- and intra-reader variability of TL measurement (especially for ill-defined lesions) may lead to a misclassification of response [5] (Fig. 3). Other similar problems occur with the choice of TL, the qualitative interpretation of the response of NTL, and the identification/interpretation of potential new lesions [3, 9]. Furthermore, in certain tumors, the implementation of loco-regional therapies and novel anticancer treatments (e.g., non-cytotoxic agents and immunotherapies) can result in structural changes within the neoplastic tissue, such as the development of necrosis, presence of inflammatory tissue, cavitation, and alterations in vascularization, which are not necessarily correlated with dimensional changes. Hence, there is a need to establish new criteria that incorporate additional morphological and functional parameters capable of capturing more accurate alterations within tumor lesions as revealed by imaging.

### iRECIST

Immune modulators have been introduced as a new anticancer therapy, with the cytotoxic T-lymphocyte antigen-4 (CTLA-4) and programmed death-1 (PD-L1) as the main targets. The result is the activation of T-cells, leading to an unusual pattern of tumor response, with a possible increase in tumor size. Conventional RECIST 1.1 criteria might, therefore, be inappropriate and lead to mistaken characterization of progressive disease. Several proposals have been made to overcome this issue, including immune-related response criteria (irRC) and irRECIST. In 2017, the RECIST working group proposed new modified RECIST criteria, the so-called iRECIST, for immune-related therapies [10] (Level of Evidence Ib). Like RECIST 1.1, iRECIST is not meant to define or guide clinical practice or treatment decisions but was created to provide a consistent framework for managing data collected in clinical trials of immune-based therapies. It is recommended to use iRECIST only as an exploratory criteria, and should be performed in parallel to RECIST 1.1 when evaluating treatment efficacy in a pharmaceutical trial [10].

**Fig. 4:**
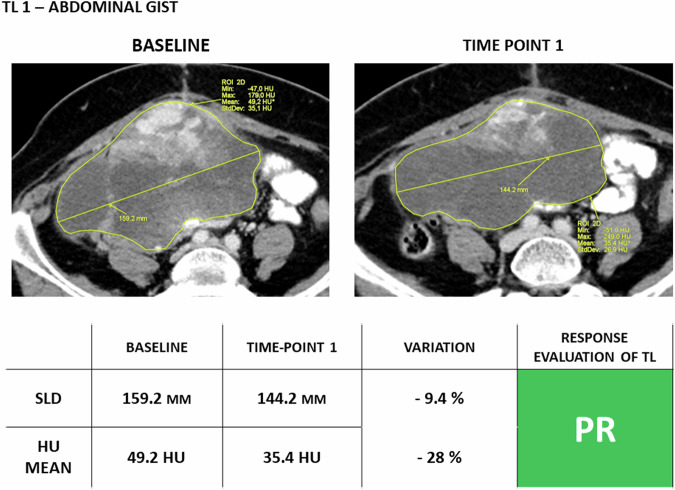
Response to treatment assessment of TL according to Choi criteria in a 68-year-old female patient with abdominal GIST. Compared to RECIST 1.1 criteria, the dimensional variation of TL is not the only parameter to evaluate, but also the HU mean must considered. On baseline, the primary tumor was selected as TL. The first time point after the start of target therapy (imatinib) showed a reduction of TL SLD insufficient to reach PR (reduction of TL SLD ≥ 10% compared to baseline), but the final response category of TL is PR because of the reduction of HU mean of 28% (cut-off for PR is a reduction of HU mean ≥ 15% compared to baseline). GIST, gastrointestinal stromal tumor; HU, Hounsfield Unit; PR, partial response; SLD, sum of the longest diameters; TL, target lesion

iRECIST is mainly based on RECIST 1.1 criteria; however, there are some important differences:

- Terminology: prefix “i” in all response evaluation nomenclature: complete response (iCR), partial response (iPR), unconfirmed progressive disease (iUPD), confirmed progressive disease (iCPD), and stable disease (iSD)
- Introduction of iUPD, aimed at overcoming the risk of misclassifying as progressive disease the increase in diameter of lesions caused by the intrinsic immune-related mechanism of action. Overall, the first progression is defined as per RECIST 1.1 criteria, but it must be confirmed and will be labeled as iUPD and must be confirmed. Three patterns of evolution may then occur: (1) In case of an increase in the number or size of any lesion and/or clinical deterioration, the progression will be confirmed (iCPD); (2) If there is no change in tumor number or size, the response remains iUPD; (3) If lesion shrinkage occurs, the iUPD will be canceled and the patient will be assessed as iSD, iPR, or iCR, and this event is known as “pseudoprogression”. Therefore, it is possible to remain at iUPD for several time points, and if there is a decrease in iSD/iPR and then progression is observed again, it would become iUPD again. This, a confirmation of progressive disease (iCPD) must follow an iUPD.

- Moreover, the response after iUPD is driven by target lesions, meaning that it is possible to have a subsequent iSD or iPR based on the sum of TL diameters even if the new lesion seen at the time of iUPD is still present or unequivocal progression in non-target lesions at the time of iUPD has not improved [11]. Overall, the iRECIST criteria include clinical status, so in case of deteriorating performance status, one could not classify the disease as pseudoprogressive. Finally, it must be noted that “pseudoprogression” is extremely rare in the real-world setting, with an estimated frequency of around 3–5% of patients, considering that the vast majority of cancer treated with immunotherapy have a radiological response similar to that to conventional chemotherapy [12, 13] (Fig. 4). In addition, new findings have suggested that the duration of response to treatment is usually shorter than the typical response, while overall survival is superior [13].

c. Assessment of new lesions: new lesions must be classified as measurable or non-measurable according to RECIST 1.1 criteria. A maximum of five new measurable lesions (maximum of two per organ) should be recorded and classified as new target lesions but should not be included in the SLD of the target lesion recorded at baseline. The remaining new measurable or non-measurable lesions should be classified as new non-target lesions. In addition, iCPD can be confirmed if new lesions appear at the next time point (4–8 weeks) or if the size of the new lesions increases compared with iUPD (sum of new target lesions ≥ 5 mm or any increase in new non-target lesions) [11].

Apart from these essential differences, the criteria closely follow RECIST 1.1: iCR/iPR are calculated from baseline, iUPD/iCPD from nadir, and the general algorithm is identical to RECIST 1.1, as well as the definitions of measurable and non-measurable lesions, site, numbers of target lesions, and response categories (Table 1).

## mRECIST criteria

Unlike most solid tumors, HCC is more commonly treated locally (with focal ablation or with chemoembolization) or with non-cytotoxic systemic therapies. The European Association for the Study of the Liver (EASL) and the American Association for the Study of Liver Diseases (AASLD) recognized the difficulties of applying WHO and RECIST criteria and promoted the development of new dedicated criteria. Thus, in 2010, Llovet and Lencioni proposed the modified RECIST criteria (mRECIST) [14].

The lack of lesion shrinkage, even after successful treatment, was overcome by introducing the modification that only viable tumor tissue is measured in TL, i.e., solid enhancing components in the arterial phase. Measuring the longest diameter of the viable tumor may be challenging when internal necrosis is present. The changes in viable tumor SLD reflect substantial changes in viable tumor volume: a reduction of ≥ 30% of the diameter of viable tumor has been calculated to correspond to a decrease of 65% in viable tumor volume, whereas an increase of 20% corresponds to an increase of ≥ 73% in viable tumor.

The nature of HCC and its coexistence with cirrhosis requires some additional specifications. Ascites and pleural effusion should not be considered neoplastic unless confirmed by cytology. Neoplastic portal vein thrombosis should be viewed as a non-measurable lesion due to the difficulties in performing reproducible measurements. Enlarged hilar lymph nodes, common in cirrhotic patients, should be considered pathologic only if their short axis is ≥ 20 mm, unlike lymph nodes in other locations, which will follow RECIST 1.1 guidelines. New lesions will be classified as HCC only if they are ≥ 1 cm in size and show a typical enhancement pattern.

Currently, mRECIST is proposed by guidelines and used by investigators to assess radiological endpoints in early and intermediate HCC treated with local treatments (Level of Evidence IV). For advanced HCC, both mRECIST and RECIST 1.1 are used (Table 1).

## Choi criteria

Choi criteria were developed to assess the response exclusively of gastrointestinal stromal tumors (GIST) treated with imatinib, a targeted therapy [15].

These criteria consider both the size and density of target lesions. Density is measured by drawing a region of interest on TL; then, the mean density is computed for all TL. Partial response is defined as a decrease in size of ≥ 10% or a decrease in tumor density of ≥ 15% (Table 1, Fig. 2). Progressive disease is defined as an increase in tumor size of ≥ 10% without meeting the criteria for partial response for density. Additionally, the appearance of new intratumoral nodules or the increased size of existing intratumoral nodules counts as progressive disease (Table 1). Choi criteria have been validated using time to progression (Level of Evidence II).

Their use has been suggested for assessing the treatment response of several different tumors, including soft tissue sarcoma, uterine leiomyosarcoma, endocrine tumors, and metastatic colorectal cancer, but without sufficient consistency or evidence to recommend them.

## Future developments

RECIST 1.1 has been validated in a large data warehouse and is a recognized surrogate of clinical endpoints. These criteria, however, do not account for shape changes of treated lesions nor for heterogeneity in the response of different lesions in the same patient. Incorporating other parameters into the criteria, such as functional information from PET, DCE-MRI, and DWI, has been suggested [16–18]. Volumetric measurements have also been suggested, but their added value has not yet been demonstrated [19, 20].

RECIST 1.1 and the other criteria were devised for use in clinical trials, not in routine clinical practice. However, their principles can also be applied when reporting outside clinical trials and are useful for radiologists who do not report for clinical trials.

## Summary statement

Standardization of imaging interpretation is especially important in clinical trials as a biomarker for overall survival and progression-free survival. RECIST 1.1 criteria are exclusively based on unidimensional lesion measurements, and changes in tumor size are used as surrogate imaging biomarkers to correlate with patient outcomes. The introduction of immunotherapy created the necessity of taking into account the possible increase in disease burden secondary to the immune response; this has led to the development of new criteria (iRECIST) with the new concept of unconfirmed progressive disease. HCC is typically treated with loco-regional treatments, and when treated systemically it is not with chemotherapy; specific criteria are used (mRECIST) in which the size measurements are performed only on the arterially enhancing portions of lesions. Choi criteria were devised for GIST, which takes into account both the size and density of neoplastic lesions since treatment with imatinib and similar drugs can reduce density/vascularization without significant changes in size. RECIST 1.1 and the other criteria were devised for use in clinical trials, not in routine clinical practice. However, their principles can also be applied when reporting outside of clinical trials and are useful for radiologists who do not report for clinical trials.

## Patient summary

Imaging plays a fundamental role in assessing the response to treatment in oncological patients because it provides essential information related to prognosis and survival. Specific criteria have been developed to evaluate CT and MRI in patients enrolled in clinical trials, and radiologists should be aware of them. The principles of these criteria can also be applied to reporting exams in patients not enrolled in clinical trials.

## Abbreviations

CR: Complete response
iCR: Immune complete response
iPR: Immune partial response
iSD: Immune stable disease
Non-CR/Non-PD: Non-complete response/non-progressive disease
NTL: Non-target lesions
OS: Overall survival
PD: Progressive disease
PR: Partial response
RECIST: Response evaluation criteria in solid tumor
SLD: Sum of the longest diameters
TL: Target lesions
WHO: World Health Organization
